# Impact of Surgical Margins and Adjuvant Radiotherapy on Local Recurrence and Survival in Sacral Chordoma

**DOI:** 10.3390/jcm14155464

**Published:** 2025-08-04

**Authors:** Furkan Erdoğan, Alparslan Yurtbay, Bedirhan Albayrak, Tolgahan Cengiz, Nevzat Dabak

**Affiliations:** 1 Department of Orthopedics and Traumatology, Faculty of Medicine, Ondokuz Mayis University, 55270 Samsun, Turkey; ndabak@gmail.com; 2 Department of Orthopedics and Traumatology, Faculty of Medicine, Samsun University, 55000 Samsun, Turkey; yurtbayalparslan@gmail.com (A.Y.); bedirhan.albayrak.5595@hotmail.com (B.A.); 3 Clinic of Orthopedics and Traumatology, Fatsa State Hospital, 52400 Ordu, Turkey; tolgahancengiz@hotmail.com

**Keywords:** chordoma, sacrum, surgical margins, radiotherapy, local recurrence, survival

## Abstract

**Background:** This study aimed to evaluate the impact of surgical margin status, tumor size, and adjuvant radiotherapy on local control, overall survival, and postoperative complications in patients undergoing surgery for sacral chordoma. **Methods:** This retrospective analysis included 18 patients who underwent surgical treatment for primary sacral chordoma between 2002 and 2019. The variables assessed included patient demographics, tumor size and volume, surgical margin status, adjuvant radiotherapy, local recurrence, overall survival, and postoperative complications. Survival analysis was performed using the Kaplan–Meier method, and appropriate parametric or non-parametric tests were used for group comparisons. **Results:** The cohort’s mean age was 62.6 ± 7.9 years, with a mean follow-up of 8.8 ± 4.1 years and an average tumor volume of 235 cm^3^. Negative surgical margins (R0) were achieved in 44% of patients. Local recurrence occurred in 50% of R0 cases and 83% of R2 cases. Negative surgical margins (R0) were associated with significantly lower local recurrence rates compared to R1 and R2 resections (Fisher’s exact test, *p* = 0.043), and showed a trend toward improved overall survival (*p* = 0.077). Overall survival was significantly lower in patients with tumors measuring ≥ 5 cm (*p* = 0.031). Adjuvant radiotherapy did not significantly reduce local recurrence (*p* = 0.245); however, an increase in complication rates was observed, although this association did not reach statistical significance (*p* = 0.108). Bladder dysfunction was significantly more frequent in patients undergoing S1–S2 resections (*p* = 0.036). **Conclusions:** Achieving negative surgical margins improves local control and may prolong survival. Larger tumors (≥5 cm) were associated with worse prognosis. While adjuvant RT may be considered in selected high-risk cases, its efficacy in preventing recurrence is unclear and may increase complication rates.

## 1. Introduction

Chordoma is a rare malignant tumor that arises from remnants of the embryonic notochord, with an estimated annual incidence of approximately 0.08 per 100,000 population [1]. It represents 1–4% of all primary bone tumors and most commonly affects the axial skeleton, particularly the sacrum and skull base [2]. Chordoma typically presents in adults between the fifth and seventh decades of life, showing a male predominance with a male-to-female ratio of approximately 2:1 [3]. Despite its indolent growth, chordoma is locally aggressive and often invades adjacent neural and osseous structures. Prognosis is influenced by tumor size, anatomical location, and the ability to achieve complete resection with negative margins [4]. Due to its rarity and insidious clinical course, conducting large-scale prospective studies is challenging; therefore, retrospective series are valuable for informing clinical management and outcomes.

Nearly half of chordomas are located in the sacrum and are frequently diagnosed at an advanced stage. This delayed diagnosis is often due to the tumor’s indolent progression and nonspecific symptoms, which allow it to reach large sizes and invade surrounding tissues at the time of detection [5,6]. Sacral chordomas typically present with nonspecific symptoms such as lower back or sacral pain, sciatica, neurogenic bladder, and bowel dysfunction, all of which further complicate timely diagnosis [7].

The primary treatment for sacral chordoma is surgical resection. The oncological adequacy of the resection—specifically, the surgical margin status (negative, marginal, or intralesional)—plays a critical role in determining local tumor control and long-term survival [8]. Studies have shown that patients who achieve negative surgical margins (R0) have significantly lower rates of local recurrence [9]. However, achieving wide surgical margins in sacral chordoma remains challenging due to the sacrum’s complex anatomical and neurovascular relationships [10].

In cases where adequate surgical margins cannot be achieved, additional therapeutic strategies may be considered to improve local control. One such strategy is adjuvant radiotherapy, especially in cases with a high risk of microscopic residual disease [11]. Nonetheless, the biological behavior of chordoma and its anatomical localization result in variable treatment responses, and the optimal treatment approach remains a matter of debate [12].

This study aims to evaluate the impact of surgical margin status, adjuvant radiotherapy, and anatomical factors on local recurrence, overall survival, and postoperative complications in patients who underwent surgical treatment for primary sacral chordoma.

## 2. Materials and Methods

This retrospective descriptive study included patients with primary sacral chordoma who underwent surgical treatment at our orthopedic oncology center between 2002 and 2019, with ongoing follow-up as of 2023. The inclusion criteria were as follows: (1) a histopathologically confirmed diagnosis of chordoma, (2) surgical treatment performed solely for the primary tumor, and (3) the availability of at least 12 months of consistent clinical and radiological follow-up. Patients who underwent surgery for recurrent disease, those who received only biopsy followed by palliative treatment, and those with incomplete follow-up data were excluded. A total of 18 patients who met the eligibility criteria were included. This study was conducted with the approval of the institutional ethics committee.

The following variables were retrospectively collected from patient records: age, sex, tumor size (<5 cm or ≥5 cm), tumor volume (cm^3^), sacral level of involvement (S1–S2 or S3–S5), surgical margin status (R0, R1, R2), adjuvant radiotherapy (RT) administration, local recurrence status, time to recurrence, overall survival, and the presence of postoperative complications. Postoperative complications were graded according to the Clavien–Dindo classification system to allow for standardized severity assessment. In addition, the association between sacral resection level and major complications, specifically bladder dysfunction, anal sphincter dysfunction, wound infection, and wound dehiscence, was analyzed.

Tumor volume was estimated approximately using preoperative imaging by multiplying the length × width × depth dimensions. Volume estimations represent approximations due to irregular tumor morphology. Surgical margin status was defined according to the American Joint Committee on Cancer (AJCC) classification as R0 (negative), R1 (microscopically positive), and R2 (macroscopically residual disease), based on final pathology reports.

All patients underwent surgery via either single-stage or combined anterior–posterior approaches based on tumor extent. Partial sacrectomy was performed in 14 patients and total or near-total sacrectomy in 4. Spinopelvic instrumentation was required in 3 high-level resections. A multidisciplinary tumor board made the decision regarding adjuvant RT based on surgical margin status and anatomical risk factors. All patients with R1 and R2 margins received adjuvant RT. In the R0 group, RT was applied selectively in cases with high-risk features such as tumor size ≥5 cm or proximity to critical neural structures. Therefore, prognostic homogeneity could not be ensured between RT and non-RT patients in the R0 subgroup.

Adjuvant RT was administered using either three-dimensional conformal radiotherapy (3D-CRT) or intensity-modulated radiotherapy (IMRT). Treatment typically began 6–8 weeks postoperatively. The radiation dose ranged from 66 to 72 Gy, delivered in daily fractions of 2 Gy, targeting the tumor bed. Minor protocol variations occurred over time due to technological advancements.

Postoperative follow-up included clinical and radiological assessments every three months during the first two years and every six months thereafter. Lumbosacral magnetic resonance imaging (MRI) was performed at each visit, and thoracic computed tomography (CT) was conducted annually for systemic metastasis screening. Local recurrence was defined radiologically as the appearance or enlargement of a mass lesion. Overall survival was calculated from the surgery date to either the date of death or last follow-up.

### Statistical Analysis

All statistical analyses were performed using SPSS version 25.0. Overall survival and local recurrence were assessed using the Kaplan–Meier method and compared with the log-rank test. Pearson’s chi-square or Fisher’s exact test was applied for categorical variables, depending on the distribution. Multivariate analysis was not performed due to the limited sample size and insufficient number of events per variable. A *p*-value of less than 0.05 was considered statistically significant.

## 3. Results

Eighteen patients (twelve males, six females) were included. The mean age was 62.6 ± 7.9 years, and the mean follow-up duration was 8.8 ± 4.1 years. Tumor localization was at S1–S2 in ten patients and S3–S5 in eight patients. The average tumor volume was 235 cm^3^. The mean overall survival was 8.6 years (95% CI: 6.7–10.6) (Table 1). The one-, five-, and ten-year overall survival rates were 94%, 67%, and 44%, respectively (Table 2). Although overall survival did not differ significantly between surgical margin groups (log-rank test, *p* = 0.077), the R0 group showed a trend toward longer median survival. In contrast, patients with negative margins had significantly lower recurrence rates than R1 and R2 groups (Fisher’s exact test, *p* = 0.043).

Tumor size was ≥5 cm in fourteen patients (78%). Kaplan–Meier analysis showed significantly reduced overall survival in patients with tumors ≥ 5 cm (*p* = 0.031), while no significant association was found between tumor volume and overall survival (*p* = 0.653). Surgical margins were classified as R0 in eight patients (44%), R1 in four patients (22%), and R2 in six patients (33%). In our series, adjuvant radiotherapy was administered in fifteen patients (83%), including five from the R0 group, four from the R1 group, and all six from the R2 group.

**Figure 1:**
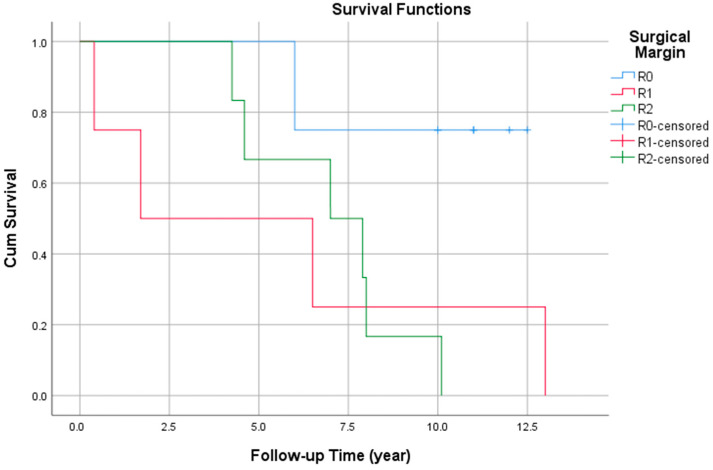
Kaplan–Meier survival curves demonstrating the association between surgical margin status and local recurrence.

Local recurrence occurred in twelve patients (67%) during follow-up. Recurrence rates were 50% (four of eight) in the R0 group, 75% (three of four) in the R1 group, and 83% (five of six) in the R2 group A statistically significant association was observed between surgical margin status and local recurrence (log-rank test, *p* = 0.043) (Figure 1). Moreover, local recurrence was significantly associated with decreased overall survival (log-rank test, *p* = 0.002).

No significant difference in recurrence rates was observed between patients who received RT and those who did not (Fisher’s exact test, *p* = 0.245). It is important to note that the non-randomized, risk-adapted nature of RT administration limits direct comparisons between these groups. Metastatic disease was detected in three patients (17%): two had gluteal muscle involvement considered likely due to local implantation following R2 resection, and one patient developed pulmonary metastasis. All metastatic patients also experienced local recurrence, suggesting a possible association between local control failure and systemic dissemination. No significant association was found between RT and metastasis (*p* = 0.317) (Table 3).

Postoperative complications occurred in seventeen patients (94%). Wound infection was observed in four patients (22%), skin necrosis in one patient (6%), and wound dehiscence in three patients (17%). Neurogenic bladder dysfunction was documented in seventeen patients (94%), with fourteen cases (82%) being permanent and three (18%) resolving within twelve months. Anal sphincter dysfunction occurred in sixteen patients (89%). To enable a standardized assessment of complication severity, all complications were classified according to the Clavien–Dindo grading system and are presented in Table 4 [13]. A statistically significant association was found between sacral resection level and bladder dysfunction, which was more common in patients with S1–S2 involvement (Fisher’s exact test, *p* = 0.036). No significant associations were observed between sacral level and other complications, including anal sphincter dysfunction, wound infection, wound dehiscence, or overall complication rates (*p* > 0.05) (Table 4).

When stratified by margin status, complication rates were 100% in the R2 group, 75% in the R1 group, and 63% in the R0 group. Among those who received RT, thirteen of fifteen patients (87%) experienced complications, compared to only one of three (33%) in the non-RT group. No statistically significant difference in complication rates was found between the RT and non-RT groups using Fisher’s exact test (*p* = 0.108).

## 4. Discussion

This study evaluated the impact of surgical margin status, tumor size, and adjuvant radiotherapy on clinical outcomes in patients undergoing surgery for primary sacral chordoma. A significant association was found between surgical margin status and local recurrence (*p* = 0.043), while overall survival was significantly lower in patients with tumors ≥ 5 cm (*p* = 0.031). No significant associations were observed between surgical margins and overall survival (*p* = 0.077) or between adjuvant radiotherapy and local recurrence (*p* = 0.245). Additionally, bladder dysfunction—an important functional outcome—was significantly more frequent in patients with S1–S2 level resections (*p* = 0.036), highlighting the role of sacral level in determining postoperative morbidity.

Consistent with Bergh et al. (2000), our findings support the importance of achieving negative surgical margins for local tumor control in sacral chordoma [14]. In our cohort, the recurrence rate was 50% in the R0 group and 67% among patients with positive margins, with a statistically significant association observed in categorical analysis (p = 0.043). These findings are in line with previous larger series: Ruggieri et al. reported significantly higher 10-year local recurrence-free survival in patients with wide negative margins (65% vs. 52%, *p* = 0.045) [12]; Ji et al. identified both tumor size and margin status as significant predictors of 5-year disease-free survival in 115 patients (*p* = 0.01) [11]; and Kiss-Bodolay et al. demonstrated superior 5- and 10-year overall survival rates (79% and 59%, respectively) in 101 patients who underwent en bloc resection without capsule violation [15].

Although margin status did not reach statistical significance for overall survival (log-rank test, *p* = 0.077), patients in the R0 group exhibited a longer median survival, suggesting a clinically relevant trend. This aligns with the prior literature: Angelini et al. (2015) and Radaelli et al. (2020) reported five-year survival rates of 72–74% in R0 patients versus ~40–45% in those with positive margins [6,10]. Given our small sample size, the lack of significance may reflect insufficient power rather than the absence of effect.

The adverse impact of larger tumor size on survival observed in our study (*p* = 0.031) is supported by York et al. (1999) and Palthe et al. (2019), who found reduced margin negativity and higher recurrence rates in tumors >9–10 cm [16,17]. Similarly, Garofalo et al. (2015) and Sciubba et al. (2009) emphasized the difficulty of achieving adequate resection in larger tumors [8,18]. Interestingly, no association was found between tumor volume and survival or recurrence in our series, likely reflecting the approximate nature of volume estimates and the limited reliability of volumetric measurements in irregularly shaped tumors.

The role of adjuvant RT in sacral chordoma remains controversial. Studies such as that by Stacchiotti et al. (2017) suggest benefit in R1/R2 resections, but the inherently low radiosensitivity of chordoma limits its efficacy (Walcott et al., 2012) [3,19]. On the other hand, DeLaney et al. (2014) reported that high-dose photon/proton radiotherapy achieved local control rates of up to 94% in primary cases [20]. In our series, the local recurrence rate was higher in patients who received adjuvant radiotherapy (60% vs. 33%), although this difference was not statistically significant (*p* = 0.245). This likely reflects selection bias, as radiotherapy was generally administered to patients with unfavorable prognostic features, such as positive surgical margins or large tumor volumes. Furthermore, only five patients in the R0 group received radiotherapy, and this subgroup was heterogeneous in terms of tumor size and anatomical risk. Therefore, interpretations regarding the efficacy of radiotherapy should be made cautiously and considered within the context of limited clinical observations.

It is essential to consider that adjuvant radiotherapy, while potentially beneficial for local tumor control, may also introduce technical challenges for subsequent surgical procedures. Radiation-induced local fibrosis, vascular compromise, and tissue adhesions can impair wound healing and make dissection more difficult in reoperations or delayed reconstructions [21]. These fibrotic changes may increase the risk of postoperative complications such as wound dehiscence, infection, or delayed recovery [22]. Although neurological symptoms were the most common postoperative complications overall, wound-related issues appeared to be more frequently associated with RT. In our series, 7 of 8 patients in the RT group developed wound complications, compared to only 1 of 3 patients in the non-RT group. This finding suggests a clinically relevant impact of RT-induced local tissue changes on wound healing. Although the difference in total complication rates between RT and non-RT groups did not reach statistical significance (87% vs. 33%; *p* = 0.108, Fisher’s exact test), the discrepancy in wound-related complications may reflect a meaningful clinical trend. Previous studies (e.g., Uhl et al., 2014) have also emphasized the adverse effects of radiation on tissue repair and neurological function [20,23]. These observations highlight the importance of individualized RT planning, particularly in anatomically challenging cases where the balance between oncologic efficacy and surgical morbidity must be carefully considered.

All three patients who developed metastases in our study also experienced local recurrence. This supports reports from Akiyama et al. (2018), Zuckerman et al. (2021), and Stacchiotti et al. (2017), which suggest that the loss of local control may predispose patients to systemic progression [19,24,25]. The involvement of the gluteal muscle in two patients likely represents local implantation following R2 resection, emphasizing the importance of achieving adequate surgical margins to minimize the risk of local tumor seeding.

Functional outcomes were closely tied to the level of sacral resection. We observed higher rates of neurogenic bladder and sphincter dysfunction in resections proximal to S3. Specifically, bladder dysfunction was significantly more common in patients with S1–S2 involvement compared to S3–S5 (Fisher’s exact test, *p* = 0.036). These results are in line with Ji et al. (2017), Sharma et al. (2021), and Sciubba et al. (2009), who emphasized the importance of preserving the S3 nerve roots in maintaining continence [8,11,26]. In our series, all patients with S1–S2 involvement developed permanent bladder and anal incontinence, underscoring the critical functional threshold of this anatomical level. Due to their permanent nature and long-term impact, these complications were classified as Grade IIIb according to the Clavien–Dindo system.

This study has several limitations. First, due to the rarity of sacral chordoma, the overall sample size was limited, which reduced statistical power, particularly for subgroup analyses. Second, the retrospective and single-center design may have introduced selection bias, especially regarding the indication and timing of adjuvant radiotherapy. Third, radiotherapy was administered in a non-randomized, risk-adapted manner; this led to heterogeneity between patient groups and made it difficult to evaluate the effect of RT in specific subgroups reliably. Finally, the long study period may have introduced variability in surgical and radiotherapy protocols over time.

## 5. Conclusions

Negative surgical margins and smaller tumor size (<5 cm) are associated with better oncologic outcomes in sacral chordoma. Adjuvant radiotherapy may be considered in selected high-risk cases; however, its effectiveness in reducing recurrence remains unclear and may increase the risk of complications. Therefore, achieving complete resection should be the primary treatment goal. Prospective, controlled studies are needed to clarify the long-term impact of radiotherapy.

## Figures and Tables

**Table 1:** Baseline demographics and tumor characteristics of the study cohort.

Characteristic	Value
Male/Female	12/6
Mean Age (±SD)	62.6 ± 7.9 years
Mean Follow-Up Duration (±SD)	8.8 ± 4.1 years
Tumor Localization (S1–S2/S3–S5)	10/8
Tumor Size ≥ 5 cm	14 (77.8%)
Mean Tumor Volume (±SD)	235 cm^3^
Surgical Margin (R0/R1/R2)	8/4/6
Partial/Total Sacrectomy	14/4
Adjuvant Radiotherapy (Yes/No)	15/3

**Table 2:** Overall survival and disease control outcomes.

Characteristic	Value
Mean Overall Survival	8.6 years (95% CI: 6.7–10.6)
1/5/10-Year Overall Survival Rates	94.4%/66.7%/44.4%
Number of Patients with Local Recurrence	12 (66.7%)
Mean Local Recurrence-Free Survival	5.96 years (95% CI: 3.79–8.14)
Number of Patients with Metastasis	3 (16.7%)

**Table 3:** Statistical association between clinical variables and outcomes.

Clinical Variables	Statistical Test	*p*-Value
Surgical margin status vs. Overall survival	Log-rank test	0.077
Tumor size (≥5 cm) vs. Overall survival	Log-rank test	0.031
Surgical margin status vs. Local recurrence	Fisher’s exact test	0.043
Local recurrence vs. Overall survival	Log-rank test	0.002
Radiotherapy vs. Local recurrence	Fisher’s exact test	0.245
Radiotherapy vs. Complication rate	Fisher’s exact test	0.108
Radiotherapy vs. Metastasis	Fisher’s exact test	0.317

**Table 4:** Postoperative complications by sacral level and Clavien–Dindo classification.

Complications	Total (n/18)	Clavien–Dindo Grade	S1–S2 (n)	S3–S5 (n)	*p*-Value
Total complications	17/18	—	11	6	0.275
Wound infection	4/18	Grade II	3	1	0.314
Skin necrosis	1/18	Grade IIIb	1	0	—
Wound dehiscence	3/18	Grade IIIa	2	1	1.000
Temporary neurogenic bladder	3/18	Grade I	1	2	—
Permanent neurogenic bladder	14/18	Grade IIIb	10	4	0.036
Temporary anal sphincter dysfunction	2/18	Grade I	1	1	—
Permanent anal sphincter dysfunction	14/18	Grade IIIb	9	5	0.630

## Data Availability

The data supporting this study’s findings are available from the corresponding author upon request.
